# The Effects of Ursolic Acid Treatment on Immunopathogenesis Following *Mannheimia haemolytica* Infections

**DOI:** 10.3389/fvets.2021.782872

**Published:** 2021-11-18

**Authors:** Jamison R. Slate, Bradley O. Chriswell, Robert E. Briggs, Jodi L. McGill

**Affiliations:** ^1^Department of Veterinary Microbiology and Preventive Medicine, Iowa State University, Ames, IA, United States; ^2^Agricultural Research Service, United States Department of Agriculture, National Animal Disease Center, Ames, IA, United States

**Keywords:** *Mannheimia haemolytica*, bovine respiratory disease complex, innate immunity, inflammation, interleukin-17A, immunomodulation, ursolic acid

## Abstract

Bovine respiratory disease complex (BRDC) is a costly economic and health burden for the dairy and feedlot cattle industries. BRDC is a multifactorial disease, often involving viral and bacterial pathogens, which makes it difficult to effectively treat or vaccinate against. *Mannheimia haemolytica* (MH) are common commensal bacteria found in the nasopharynx of healthy cattle; however, following environmental and immunological stressors, these bacteria can rapidly proliferate and spread to the lower respiratory tract, giving rise to pneumonic disease. Severe MH infections are often characterized by leukocyte infiltration and dysregulated inflammatory responses in the lungs. IL-17A is thought to play a key role in this inflammatory response by inducing neutrophilia, activating innate and adaptive immune cells, and further exacerbating lung congestion. Herein, we used a small molecule inhibitor, ursolic acid (UA), to suppress IL-17A production and to determine the downstream impact on the immune response and disease severity following MH infection in calves. We hypothesized that altering IL-17A signaling during MH infections may have therapeutic effects by reducing immune-mediated lung inflammation and improving disease outcome. Two independent studies were performed (Study 1 = 32 animals and Study 2 = 16 animals) using 4-week-old male Holstein calves, which were divided into 4 treatment group including: (1) non-treated and non-challenged, (2) non-treated and MH-challenged, (3) UA-treated and non-challenged, and (4) UA-treated and MH-challenged. Based on the combined studies, we observed a tendency (*p* = 0.0605) toward reduced bacterial burdens in the lungs of UA-treated animals, but did not note a significant difference in gross (*p* = 0.3343) or microscopic (*p* = 0.1917) pathology scores in the lungs. UA treatment altered the inflammatory environment in the lung tissues following MH infection, reducing the expression of IL-17A (*p* = 0.0870), inflammatory IL-6 (*p* = 0.0209), and STAT3 (*p* = 0.0205) compared to controls. This reduction in IL-17A signaling also appeared to alter the downstream expression of genes associated with innate defenses (BAC5, DEFB1, and MUC5AC) and lung remodeling (MMP9 and TIMP-1). Taken together, these results support our hypothesis that IL-17A signaling may contribute to lung immunopathology following MH infections, and further understanding of this inflammatory pathway could expand therapeutic intervention strategies for managing BRDC.

## Introduction

Bovine respiratory disease complex (BRDC) is a leading cause of morbidity and mortality within the North American feedlot (1) and dairy (2) industries, and its economic burden is estimated to cost in excess of 1 billion dollars annually due to increased labor and production-loss expenses (3, 4). Current vaccinations strategies are limited in their efficacy (5), and BRDC is often difficult to treat because it is a multifactorial disease. These infections typically involve a combination of environmental and host factors, as well as multiple viral and bacterial pathogens. Unfortunately, many BRDC pathogens are highly pervasive, and early production practices, like weaning and shipping, can leave young cattle immunologically stressed and susceptible to primary infections. *Mannheimia haemolytica* (MH) is a common commensal bacteria found in the nasopharynx of healthy cattle, where its proliferation is generally limited by other commensal flora and the host's immune system (3, 4). However, following immunological stressors or a primary viral infection, MH can migrate deeper into the lower respiratory tract, eventually culminating in severe bacterial pneumonia (4). Although BRDC is a syndrome involving multiple viral and bacterial pathogens, MH is often the most prevalent bacteria isolated from pneumonic bovine lungs, and it is considered the principal bacterial agent involved in BRDC (6). Severe MH infections are often characterized by fibrinous exudate and an accumulation of activated macrophages and neutrophils, which further contribute to lung congestion through inflammatory cytokine signaling and excretion of cytotoxic compounds (6, 7).

Interleukin-17A (IL-17A) is an inflammatory cytokine known to aid in the infiltration and activation of neutrophils, and recent studies have shown that MH infections induce an upregulation of IL-17A expression in the lungs (8). Our recent studies have shown that IL-17A is also upregulated in the lungs of calves infected with bovine respiratory syncytial virus (BRSV), a common viral agent associated with BRDC. Inhibiting IL-17A production during BRSV infection was shown to reduce disease severity and lung damage in neonatal calves (9). Considering that severe cases of MH infection are often associated with increased lung inflammation and cytotoxic neutrophil infiltration (10, 11), IL-17A signaling following MH infection may be contributing to immune-mediated tissue damage and pathogenesis (8). Therefore, we hypothesized that inhibiting IL-17A signaling during MH infection may have beneficial effects on immune-mediated lung inflammation and disease outcome.

Previous studies in our lab have used a small molecule drug, digoxin, to inhibit the production of IL-17A in neonatal calves; however, digoxin treatment can have considerable cytotoxic effects, limiting its potential as a therapeutic inhibitor of IL-17A (8, 9). Recent research has suggested that another small molecule drug, ursolic acid (UA), may have similar inhibitory capabilities with fewer cytotoxic effects (12, 13). Herein, we confirmed that UA treatment can reduce IL-17A production at non-cytotoxic levels using bovine peripheral blood mononuclear cells (PBMCs). We subsequently employed prophylactic UA treatment as an *in vivo* tool to disrupt IL-17A signaling following MH infection of neonatal calves.

UA-treated calves showed a favorable trend toward reduced bacterial burdens in the lungs compared to control calves. Additionally, UA treatment reduced IL-17A expression *in vivo* in pneumonic lungs, and also significantly reduced expression of inflammatory interleukin-6 (IL-6) and the regulatory transcription factor, signal transducer and activator of transcription 3 (STAT3). Interestingly, untreated control calves had a higher expression ratio of matrix metalloproteinase 9 (MMP9) to tissue inhibitor of metalloproteinase 1 (TIMP-1) in their lungs compared to UA-treated calves, which indicates ongoing extracellular matrix destruction is not being dampened for tissue repair and remodeling. This result is consistent with the increased inflammatory response and more severe lung scores observed in control animals compared to UA-treated calves. Taken together, our results suggest that inhibiting inflammatory IL-17A signaling may decrease lung bacterial burden and alter host immune responses to potentially reduce disease severity following MH infection. By further exploring the role of inflammatory IL-17A signaling in MH infections, we hope to expand therapeutic intervention strategies for severe respiratory infections that contribute to the pathogenesis of BRDC.

## Materials and Methods

### MTT Cell Viability Assay

The CyQUANT MTT Cell Viability Assay (Invitrogen) was used according to manufacturer's recommendations. Varying concentrations of UA (AstaTech) in DMSO were prepared using serial dilutions, and 5 μg/ml of Concanavalin A (ConA, MP Biomedicals) in complete RPMI media (cRPMI) was prepared for cellular stimulation. The cRPMI was composed of RPMI-1640 (Gibco) supplemented with 2 mM L-glutamine, 25 mM HEPES buffer, 1% antibiotic-antimycotic solution, 1% sodium pyruvate, 1% non-essential amino acids, 2% essential amino acids, 50 μM 2-mercaptoethanol, and 10% (volume/volume) fetal bovine serum as outlined in McGill et al. (14). Bovine PBMCs were suspended in cRPMI and plated into 96-well round-bottomed plates at 250,000 cells per well, and then corresponding UA, ConA, DMSO (carrier control), and cRPMI solutions were added to the wells for a total volume of 200 μl in each well. Cells were incubated in the dark at 37°C and 5% CO_2_ for 72 h. Cell cultures were then centrifuged to pellet the cells and supernatants were collected for ELISA analysis. Cells were re-suspended in the CyQUANT MTT reagent and then returned to the incubator for an additional 4 h. MTT crystals were vigorously mixed with SDS-HCl solubilization mixture and returned to the incubator for another 4–8 h. The solubilized MTT mixture was transferred to a flat-bottomed 96-well plate for microplate spectrophotometer analysis at 570 nm.

### ELISA for IL-17A Production

IL-17A production by bovine PBMCs was determined using the Bovine IL-17A Do-It-Yourself ELISA kit (Kingfisher Biotech, Inc) following the manufacturer's published ELISA Technical Guide (Kingfisher Biotech, Inc). In summary, anti-bovine IL-17A capture antibody was coated overnight at 4°C in 96-well plates (Themo Scientific MaxiSorp, Immulon 4 HBX) at a concentration of 2.5 μg/ml. Plates were then blocked with a 4% bovine serum albumin in PBS solution for 1 h at room temperature. Plates were washed with a 0.05% Tween 20 in PBS buffer, and bovine IL-17A standard or stimulated PBMC cell culture supernatants (from the MTT cell viability assays) were incubated at room temperature for 2 h. The plates were washed and then incubated with biotinylated anti-bovine IL-17A detection antibody at a concentration of 0.1 μg/ml for 1 h at room temperature. The plates were washed and then incubated with 1:100 HRP-Streptavidin (Kingfisher Biotech) in assay diluent for an additional hour at room temperature. Plates were washed a final time, and then incubated with 1-Step Ultra TMB ELISA Substrate (ThermoFisher Scientific) until sufficient color change was detected in the standards. The reaction was then stopped with 2 M H_2_SO_4_ stop solution and the sample absorbance was measured at 450 nm using an ELISA microplate reader (Fisher Scientific accuSkan™ FC Filter-Based Microplate Photometer).

### Animal Husbandry

All animal procedures were conducted in accordance with institutional guidelines, and protocols were approved by Iowa State University's Institutional Animal Care and Use Committee (IACUC-19-081).

The fall 2019 (F2019) study enrolled 32, male, 4-week-old Holstein and Holstein/cross calves which were randomly divided into 4 treatment groups (*n* = 8 animals per group): non-treated and non-challenged, UA-treated and non-challenged, non-treated and MH-challenged, and UA-treated and MH-challenged. The summer 2020 (S2020) study enrolled 16 male Holstein and Holstein/cross calves, all ~4 weeks old at the time of receiving, which were randomly divided into 2 treatment groups (*n* = 8 animals per group): non-treated and MH-challenged, and UA-treated and MH-challenged. Calves were housed by treatment group, with 4 animals per room, under climate controlled ABSL2 conditions in the Livestock Infectious Disease and Isolation Facility at Iowa State University.

Animals had *ad libitum* access to water, hay, and calf starter. Calves were fed 3 quarts of milk twice daily (once in the morning and once in the evening) in buckets for the duration of the study. Beginning 3 days prior to MH infection and for the remainder of the trial, UA-treated animals received 0.5 g of UA mixed into their milk at each feeding, resulting in a total of 1 gram of UA powder per day. The selection of UA was determined based on the doses used in the literature for mice and allometric scaling calculations (12, 13, 15, 16); the equation and theories for these allometric scaling calculations have been previously summarized in West and Brown (17).

### MH Intratracheal Infection

Intratracheal inoculation was performed as previously described (18). F2019 animals were challenged intratracheally with 4.15 × 10^8^ of MH (D153 and D174 strains) in 50 ml of Earle's balanced salt solution (EBSS), followed by a 50 ml intratracheal wash of EBSS: the MH inoculation mixture was comprised of 2.05 × 10^8^ of MH strain D153 and 2.10 × 10^8^ of MH strain D174 suspended in EBSS. F2020 animals were challenged intratracheally with 1.98 × 10^8^ of MH (D153 and D174 strains) in 50 ml of EBSS, followed by a 50 ml intratracheal wash of EBSS: the MH inoculation mixture was comprised of 1.05 × 10^8^ of strain D153 and 9.25 × 10^7^ of strain D174 suspended in EBSS.

### Clinical Scoring

Calves were monitored for signs of clinical illness and scored every day beginning on the day of MH infection, or 0 days post infection (0 dpi). Calves were scored using a modified University of Wisconsin Calf Health Respiratory Scoring Chart (9). Scoring categories include nasal discharge, eye discharge, ear tilt, spontaneous or handler-induced coughing, lung sounds detected by auscultation, expiratory effort and rectal temperature (**0**, temperature is <101.0°F; **1**, temperature is 101.1–101.9°F; **2**, temperature is 102.0–102.9°F; **3**, temperature is ≥103.0°F).

The study protocols included provisions for a humane endpoint. Eleven calves from the F2019 study were euthanized early after reaching a humane endpoint: 3 animals in the non-treated and non-challenged group, 0 animals in the UA-treated and non-challenged group, 5 animals in the non-treated and MH-challenged group, and 3 animals in the UA-treated and MH-challenged group. Two calves from the S2020 study were euthanized after reaching a humane endpoint. This included 1 calf from the non-treated and MH-challenged group, which was euthanized before MH infection, and 1 calf from the UA-treated and MH-challenged group, which was euthanized within 24 h of MH infection.

### Sample Collections

Nasal swabs were collected by swabbing both nostrils with sterile polyester-tipped applicators (Puritan) on three separate occasions through the course of each study; 3 days prior to infection (−3 dpi), on the day of MH infection but before intratracheal inoculation (0 dpi), and at the time of necropsy (4 dpi, or humane endpoint).

Blood and serum samples were collected through the jugular vein; whole blood for immediate use in assays was collected in acid citrate dextrose (ACD, yellow-top) tubes, while blood for serum isolation was collected in silica clot activator (marbled red-gray-top) tubes. Blood collected in serum tubes was allowed to clot, centrifuged for serum separation, and aliquoted for storage in −80°C.

### Necropsy

Calves were euthanized by barbiturate overdose and necropsied on 4 dpi or upon reaching humane endpoint. At necropsy, the lungs of each calf were cleaned, photographed, and scored based on the observable lung consolidation and lesion area. The lungs were divided into nine sections and each section was visually evaluated and palpated for the degree of pneumonic consolidation. Scored sections included the cranial half of left cranial, caudal half of left cranial, left caudal, accessory, cranial half of right cranial, caudal half of right cranial, right middle, and right caudal. These sectional scores were then weighted based on air exchange: cranial half of left cranial (4%), caudal half of left cranial (6%), left caudal (32%), accessory (4%), cranial half of right cranial (6%), caudal half of right cranial (5%), right middle (7%), and right caudal (35%) and each lung was assigned a final score (18).

Bronchoalveolar lavage (BAL) fluid was collected at the time of necropsy. A total of 500 mL of ice-cold saline containing 1% antibiotic-antimycotic solution was washed through the trachea and massaged into the lungs. The BAL fluid was then transferred into sterile collection bottles and stored on ice before processing in the lab. BAL fluid was filtered through sterile gauze, and aliquots were submitted for cytospin and cytology analysis at the Iowa State University Veterinary Diagnostic Laboratory. The remaining BAL fluid was centrifuged and washed twice with cold (4°C) PBS, and then live cells were enumerated using trypan blue exclusion. Cells were suspended in cRPMI for cellular assays or freezing media (10% DMSO in FBS) for storage in liquid nitrogen.

Representative tissue sections were excised from the trachea and lungs (right cranial, upper-right caudal, lower-right caudal, lower-left caudal, upper-left caudal, left cranial, and accessory) of each animal. Samples were fixed in formalin and submitted to the Iowa State University Veterinary Diagnostic Lab for pathological evaluation by a certified veterinary pathologist, stored in either Qiagen RNAlater solution for quantitative polymerase chain reaction (qPCR) analysis, or snap frozen over dry ice for bacterial quantification. Samples of liver and spleen were also snap frozen for bacterial quantification. All samples were stored at −80°C until analysis.

### Bacterial Recovery

Bacterial lung loads were determined by quantitative culture and reported as total colony forming units (CFUs) as previously described (18). Briefly, lung tissue samples were ground in EBSS to produce a homogenized suspension that was then diluted 10-fold in EBSS. The dilutions were then spread on blood agar base plates containing 5% defibrinated bovine blood and incubated overnight at 37°C. Colonies with typical MH morphology were enumerated, and representative colonies were selected for plate agglutination. Swabs were rolled on half of a fresh blood agar plate, then a sterile loop was used to semi-quantitatively streak for isolation on the remaining two quarters. In addition to rapid plate agglutination (19), PCR analyses were performed. In MH, primers targeting Lkt D (all MH) and capsule specific regions (MH st1 and MH st6) were used to confirm the lung isolates produced the same PCR products as the challenge strains (Briggs, unpublished-sequences available upon request). In Pasteurella, KMT primers were used (20). Isolates were sent to NVSL diagnostics for MALDI-ToF identification.

### Dihydrorhodamine 123 (DHR) Reactive Oxygen Species Assay

Approximately 3 mL of fresh whole blood collected in ACD tubes was transferred to a 15 mL conical. Each tube of whole blood was volumed up to the top with warm (35°C) red blood cell lysis buffer and incubated in a 35°C water bath for 5 min, agitating the tubes halfway through the incubation. Cells were centrifuged and washed with warm PBS, then re-suspended in warm Hanks' balanced salt solution (Gibco, 14175-095) and plated in 96-well round-bottomed plates. The cells were re-suspended in a 75 μM solution of DHR (FITC+) in HBSS and incubated at 37°C for 20 min. After 20 min, cells were stimulated with 100 ng of PMA and returned to the incubator for an additional 25 min. Following incubation, the cells were placed on ice for 5 min, centrifuged, and re-suspended in FACS buffer (10% fetal bovine serum, 0.1% sodium azide, and PBS). The cells were surface stained with 10 μg/mL mouse anti-bovine CD14 to detect monocytes (clone CAM36A, IgG1, Kingfisher Biotech, Inc.) and mouse-anti-bovine granulocytes to detect neutrophils (clone CH138A, IgM, Kingfisher Biotech, Inc.). Cells were incubated for 30 min at 4°C. Cells were washed 2 times with FACS buffer and stained with 5 μg/mL mouse anti-IgG1-PE (BD Pharmingen) and goat anti-mouse IgM-AF647 (Invitrogen) for 30 min at 4°C. Finally, cells were fixed in 1x BD FACS lysing solution (BD Bioscience) at room temperature for 10 min and stored in FACS buffer for flow cytometry analysis. Supplementary Figure 1 includes a representative FACS gating strategy. Flow cytometry data were collected on BD FACS Canto II (BD Biosciences) and analyzed using FlowJo software version 10.6.1 (BD Biosciences).

### RT-qPCR Analysis of Lung Tissues

Pneumonic (lesion) and healthy (non-lesion) control (non-treated, non-challenged) lung tissue samples were stored in RNAlater at −80°C until processing for PCR analysis. RNA isolation, cDNA preparation, and quantitative PCR were performed as previously reported (8). Primer sequences are compiled in Table 1. Primers for RORC, IL-8, and DEFB1 were designed using Integrated DNA Technologies' (IDT) PrimerQuest™ Tool. Primers for BAC5 were a kind gift from Dr. Corwin D. Nelson, University of Florida. Relative expression (RE) for each gene was determined using the 2^−ΔΔCT^ method (28), with RPS9 used as a reference housekeeping gene from which Δ cycle thresholds (CT) were determined. Briefly, the RE was calculated as RE = 2^−ΔΔCT^, where ΔΔCT = ΔCT of pneumonic lung – Average ΔCT of healthy control lungs. These RT-qPCR data were depicted with the Average RE of a particular gene along with the individual RE values for each animal.

**Table 1:** Primers for RT qPCR Analysis.

**Primer Table**			
**Gene target**	**NCBI reference sequence**	**Primer sequence**	**References**
RPS9	NM_001101152.2	F: 5′ CGCCTCGACCAAGAGCTGAAG 3′ R: 5′ CCTCCAGACCTCACGTTTGTTCC 3′	(21)
IL-6	NM_173923.2	F: 5′ CTGAAGCAAAAGATCGCAGATCTA 3′ R: 5′ CTCGTTTGAAGACTGCATCTTCTC 3′	(21)
STAT3	NM_001012671.2	F: 5′ GACCGGTGTCCAGTTCACAA 3′ R: 5′ AAATTTCCGGGACCCTCTGA 3′	(22)
RORC	NM_001083451.2	F: 5′ GTCAGCGCTCCAATATCTTCTC 3′ R: 5′ CTTAGCGAACTCCACCACATA 3′	
IL-17A	NM_001008412.2	F: 5′ CACAGCATGTGAGGGTCAAC 3′ R: 5′ GGTGGAGCGCTTGTGATAAT 3′	(23)
IL-8	NM_173925.2	F: 5′ CGCTGGACAGCAGAGCTCACAAG 3′ R: 5′ GCCAAGAGAGCAACAGCCAGCT 3′	
BAC5 (CATHL2)	NM_174826.3	F: 5′ TTCAAGGAGAATGGGCTGGT 3′ R: 5′ GATCGGTGGGAAGATCGGTG 3′	
DEFB1	NM_001324544.1	F: 5′ GTCAGGAATAAGTGATTTTGCAAGC 3′ R: 5′ GCCGGAAACAGATGCCAATC 3′	(24)
MUC5AC	XM_024987596.1	F: 5′ CAGACCCTCCACCTTCTTCA 3′ R: 5′ GGTCCTCGAAGCTGTTCTTG 3′	(25)
IFNγ	NM_174086.1	F: 5′ AGAATCTCTTTCGAGGCCGGAG 3′ R: 5′ TATTGCAGGCAGGAGGACCATTAC 3′	(21)
MMP9	NM_174744.2	F: 5′ GACCAGGACAAGCTCTACGG 3′ R: 5′ CAGAAGCCCCACTTCTTGTC 3′	(26)
TIMP-1	NM_174471.3	F: 5′ GATGTCGTCATCAGGGCC 3′ R: 5′ TCGCTCTGCAGTTTGCAG 3′	(27)

### Statistical Analysis

All statistical analyses were made using Prism v9.1.0 (GraphPad Software, Inc.).

The treatment means ± the standard error of the means (SEM) were plotted for the *in vitro* MTT and ELISA assays, and significant differences between these groups were determined using one-way ANOVA with Tukey's multiple comparisons. Clinical scoring data was plotted as the mean clinical score ± SEM for each treatment group, and the data was analyzed by two-way ANOVA (time and treatment) mixed-effects model with multiple comparisons. Pneumonic consolidation (lung lesion) scoring and pathology scoring data were plotted with the mean score ± SEM along with individual scores for each animal amongst the two studies; outliers were removed from these data sets using the ROUT method (Prism v9.1.0), and significant differences between treatment effects were determined using a standardized unpaired *t*-test. Similarly, bacterial burden data were reported on a log scale with mean CFUs ± SEM, as well as CFUs for each animal; outliers for these data sets were removed using the ROUT method (Prism v9.1.0) and significant differences between treatment effects were determined using a standardized unpaired *t*-test. The reactive oxygen species (DHR) assays were reported as the mean change in fluorescent intensity (ΔMFI) ± SEM, and significant differences between treatments were determined using an unpaired *t*-test. RT-qPCR data were reported as the Average RE ± SEM for each gene, and included individual REs for each animal; once again, outliers were removed using the ROUT method (Prism v9.1.0), and significant treatment differences were determined using a standard unpaired *t*-test.

## Results

### UA Treatment Inhibits IL-17A Cytokine Production by Bovine PBMCs *in vitro*

**Figure 1:**
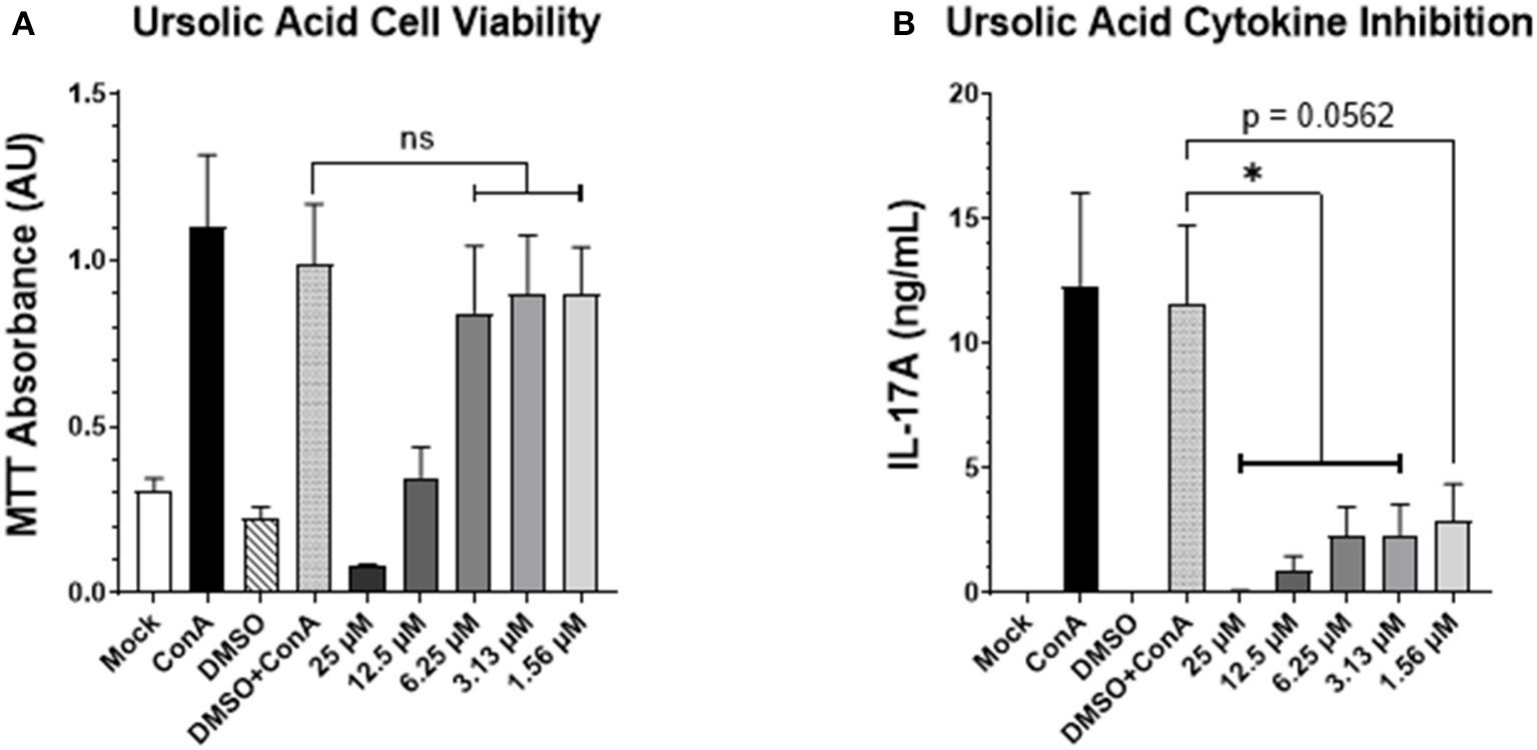
UA suppresses mitogen-induced IL-17A production by bovine PBMCs. Peripheral blood mononuclear cells from three healthy bovids were plated at 3 × 10^5^ cells per well in a 96-well plate and incubated with Concanavalin A, vehicle control (DMSO), or escalating concentrations of UA dissolved in DMSO for 3 days. On day three, cell supernatants were collected, and the remaining cell pellet was re-suspended with an MTT solution using the manufacturer's protocol to subsequently analyze the viability of the cells after the incubation period. **(A)** Mean absorbance values at 570 nm ± SEM following the MTT incubation and solubilization. Significance was determined using a one-way ANOVA with Tukey's multiple comparisons; (ns) indicates no significant differences were observed. **(B)** The supernatants from the 3-day incubation were analyzed for IL-17A concentration by ELISA. Data represent mean IL-17A concentration (in ng/mL) ± SEM detected in the supernatants. The data were analyzed using a standard one-way ANOVA with Tukey's multiple comparisons; ^*^p < 0.05.

Previous studies in our lab have shown that reducing IL-17A production using digoxin (a small molecule inhibitor of the RORγt transcription factor) can ameliorate a viral respiratory disease in cattle but with potential cytotoxic effects. However, recent investigations have shown that another small molecule inhibitor of RORγt, ursolic acid (UA), can similarly suppress IL-17A production without cytotoxic effects in mouse and human cells (12, 13). To assess the cytotoxicity and verify the inhibitory capabilities of UA within the context of a bovine model, we incubated bovine PBMCs with escalating concentrations of UA and concanavalin A (ConA) for 72 h. We then used an MTT assay to test cell viability, and an enzyme-linked immunosorbent assay (ELISA) to screen for IL-17A production in the cell supernatants. The MTT assay (Figure 1A) demonstrated that the three lowest concentrations of UA (dissolved in DMSO) were not significantly different in their absorbance values from the carrier control (DMSO + ConA), indicating no loss of cell viability due to UA treatment. However, the two highest concentrations of UA treatment (12.5 and 25 μM) did have a negative impact on cell viability compared to the carrier control (*p* = 0.0901 and 0.0064, respectively). As seen in Figure 1B, all three of the lowest UA treatment concentrations had a substantial impact on IL-17A production, with the 3.13 and 6.25 μM concentrations causing a significant reduction in cytokine production compared to the carrier control (*p* = 0.0362 and 0.0350 respectively), while the 1.56 μM UA treatment also showed notable inhibitory potential (*p* = 0.0562). Although both the 12.5 and 25 μM treatments showed significant reductions in IL-17A production (*p* = 0.0112 and 0.0057, respectively), we cannot determine if the reduced cytokine production was due to inhibitory effects from the treatment or reduced cell viability (Figure 1A). Taken together, these data show that UA treatment can effectively reduce IL-17A production at concentrations that are not cytotoxic to bovine lymphocytes *in vitro*.

### Impact of Prophylactic UA Treatment on Clinical Disease and Lung Pathology Following *in vivo* MH Infection in Calves

**Figure 2:**
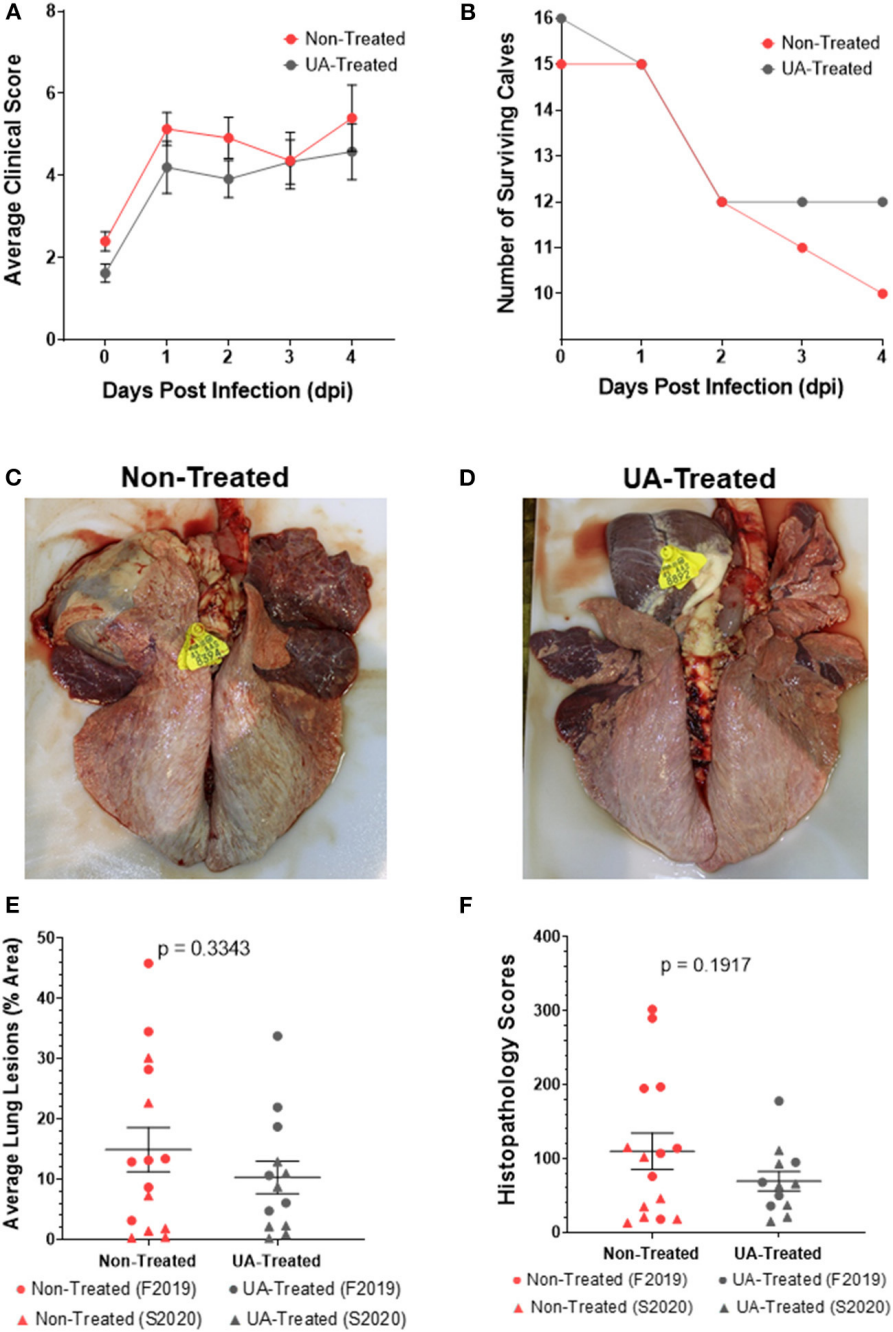
Effects of prophylactic UA treatment on clinical disease and lung lesions following *Mannheimia haemolytica* infection. In both studies (F2019 and S2020) animals were divided into non-treated and UA-treated groups (*n* = 8 calves per group). UA-treated animals were given 1 g per head per day of UA powdered supplement with milk feedings beginning 3 days before infection (-3 dpi) and through the duration of the study. Non-treated animals were given no additional supplements with their milk feedings. Animals were challenged through intratracheal instillation with *Mannheimia haemolytica* (~1 × 10^8^ each of strains D153 and D174) (0 dpi). **(A)** Calves were monitored and scored daily using a modified Wisconsin scoring system. The data depict the average daily clinical score ± SEM pooled from both studies (*n* = 15 per group). The data were analyzed by two-way ANOVA mixed-effects model with multiple comparisons. **(B)** Calves were removed from their respective studies upon reaching a humane endpoint, and the number of surviving calves in each treatment group were charted over the 4 day challenge period. **(C)** Representative image of a non-treated lung (S2020) at 4 dpi. **(D)** Representative image of a UA-treated lung (S2020) at 4 dpi. **(E)** At necropsy, lungs were photographed and scored based on the percentage of that area that was affected with pneumonic consolidation. Individual animal data from F2019 has been represented as a circle, while S2020 animal data has been represented as a triangle. This figure shows the average lung consolidation ± the SEM for each treatment group (non-treated *n* = 15, UA-treated *n* = 13); the data were analyzed using a standardized unpaired *t*-test. **(F)** Samples from each lung were collected and submitted to a blinded pathologist for scoring as described in McGill et al. (9). Individual animal data from F2019 has been represented as a circle, while S2020 animal data has been represented as a triangle. The graph depicts the mean histopathology ± SEM for each treatment group (non-treated *n* = 15, UA-treated *n* = 12). Outliers in the data were first identified using the ROUT method (Prism v9.1.0), and the data was subsequently analyzed using a standardized unpaired *t*-test to determine significant differences between the two treatments.

Based on our *in vitro* data from Figure 1, we next chose to use UA treatment to evaluate the role of IL-17A on the outcome of MH infection *in vivo* in calves. Full study details are provided in section Animal Husbandry, but in brief, a total of 48, 4-week-old, male Holstein calves were enrolled in two independent studies (F2019 *n* = 32 calves; S2020 *n* = 16 calves). Additionally, calves were either treated with 1 g/head/day of UA mixed into milk replacer, or received no treatment in their milk replacer at feedings. Treatment was initiated 72 h prior to MH infection and continued for the duration of the trials. On day 0, calves were challenged intratracheally with MH. The calves were monitored daily for clinical signs of infection until the study endpoint on 4 dpi. As seen in Figure 2A, clinical scores quickly rose within the first 24 h of infection and stabilized between 1 and 4 dpi with no significant differences between the two treatment groups. Several animals in the F2019 and S2020 cohorts reached a humane endpoint prior to 4 dpi and were removed from the trial (Figure 2B). In total, 8 animals were removed early from the F2019 study: 5 calves from the non-treated group and 3 calves from the UA-treated group. There were only 2 animals removed early from the S2020 study: 1 calf from the non-treated group was removed before the MH challenge, and 1 calf from the UA-treated group was removed shortly after MH challenge. We observed no differences in mortality between treatment groups.

Necropsies were performed on 4 dpi, or upon reaching a humane endpoint. At necropsy, lungs were photographed (Figures 2C,D), scored for consolidated lung area (Figure 2E), and tissue sections were fixed in formalin for histopathology scoring (Figure 2F). Figure 2C shows a representative image of lung consolidation from a non-treated calf. Figure 2D shows a representative image of lung consolidation from a UA-treated calf. The lung consolidation scores were combined from both studies, and the average lesion area is depicted in Figure 2E. We did not detect a statistically significant difference in lung lesions in UA-treated animals compared to controls (*p* = 0.3343); however, the mean area of pneumonic lung in UA-treated calves (10.31%) was lower than the mean area of pneumonic lung in non-treated calves (14.91%). The S2020 animals tended to cluster lower in lung lesions compared to the F2019 animals.

Histopathology scores for each individual animal are shown in Figure 2F. Histopathology scores were not significantly different between treatment groups (*p* = 0.1917); however, the mean histopathology score of UA-treated animals (69.42) was lower than the mean score of non-treated controls (109.90). Similar to the lung lesion scoring in Figure 2E, the F2019 animals tended to have the highest histopathology scores in Figure 2F compared to the S2020 animals. Taken together, the reduced means of both lung consolidation scoring and histopathology scoring suggest that IL-17A signaling may contribute to lung pathology following MH infection.

### Prophylactic UA Treatment Maintains Immune Cell Populations and Functionality While Reducing Bacterial Burden Following MH Infection

**Figure 3:**
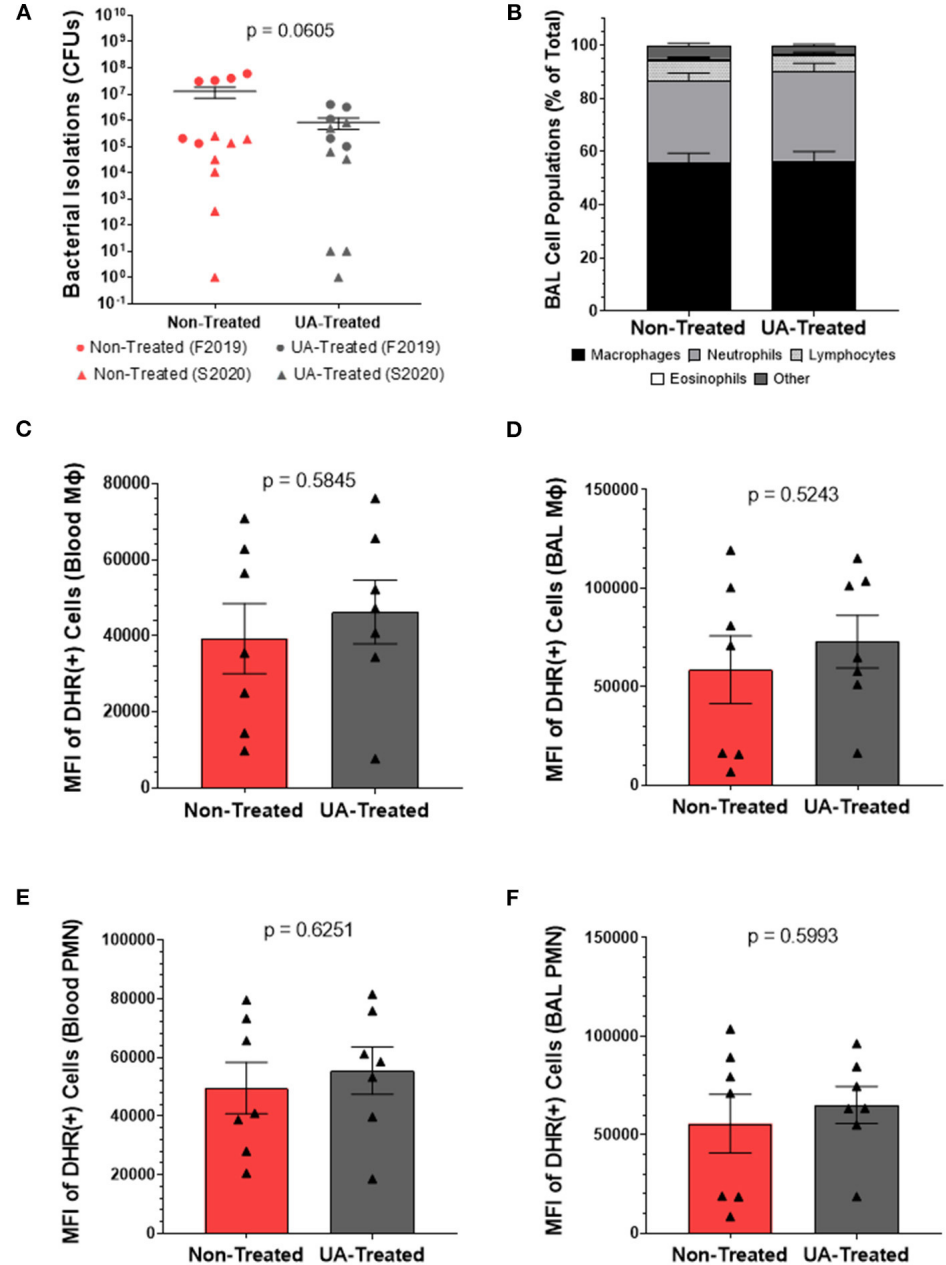
Prophylactic UA treatment reduces bacterial burden following MH infection but does not affect the oxidative burst potential of infiltrating or circulating innate immune cells. **(A)** Lung sections were collected from 7 predetermined locations around the lung and assayed by quantitative culture to determine the bacterial burden at 4 dpi as described in Briggs et al. (18). Individual animal data from F2019 has been represented as a circle, while S2020 animal data has been represented as a triangle. The average number of colony forming units (CFUs) determined from each animal are plotted on the graph above along with the average CFUs ± the SEM for each treatment group (non-treated *n* = 13, UA-treated *n* = 12). **(B)** BAL fluid was collected at 4 dpi and subjected to cytology analysis to quantify neutrophils, macrophages, lymphocytes, eosinophils, and other cell types infiltrating the lung airways. The graph depicts the mean relative frequencies of cell types ± SEM for each treatment group (non-treated *n* = 14, UA-treated *n* = 13). **(C–F)** Whole blood and BAL fluid were collected for DHR assays at 4 dpi to determine the oxidative burst potential of circulating macrophages **(C)**, BAL macrophages **(D)**, circulating neutrophils **(E)**, and BAL neutrophils **(F)** in the two treatment groups; these data were only collected during the S2020 study, thus individual animal data is represented with a triangle symbol (▴). Cells were stimulated with PMA or remained unstimulated for 30 min. Cells were then surface stained with monoclonal antibodies to detect monocytes (anti-bovine CD14) and granulocytes (anti-bovine CH138A) and fixed for flow cytometry analysis. These figures depict the average ΔMFI (MFI of PMA stimulated minus MFI of unstimulated wells) ± SEM of DHR labeled and PMA stimulated cells. Data represent animals from the S2020 study only (non-treated *n* = 7, UA-treated *n* = 7).

We next performed quantitative culture for MH on lung tissue sections collected at necropsy to determine bacterial loads in UA-treated and non-treated calves. As seen in Figure 3A, the average MH CFUs recovered by quantitative culture tended to be lower in the UA-treated animals compared to the non-treated animals (*p* = 0.0605).

Since IL-17A signaling is known to be a chemoattractant for neutrophils, we sampled the cellular populations of the lung environment by collecting BAL fluid for cytospin analysis. Figure 3B depicts the relative frequencies of macrophages, neutrophils, lymphocytes, eosinophils, and other cell types infiltrating the lung airways. We observed no significant differences in the relative proportions of these cell populations between non-treated and UA-treated animals.

We next chose to determine the impact of UA treatment on the oxidative burst capacity of neutrophils and macrophages from MH infected calves. Functional immune cells undergoing oxidative burst release reactive oxygen species (ROS) that oxidize dihydrorhodamine 123 (DHR) into fluorescent rhodamine 123; thus, the mean fluorescence intensity (MFI) reported in Figures 3C–F is a measure of ROS generated from oxidative burst in cells collected 4 dpi. This assay was conducted only on samples from the S2020 study. Figures 3C,D show ROS production generated from circulating and BAL infiltrating macrophages (CD14^+^ cells), respectively. Figures 3E,F show ROS production generated from circulating and BAL infiltrating peripheral mononuclear cells (PMNs, CH138A^+^ cells), respectively. ROS production from circulating (3C) and lung infiltrating (3D) macrophages was similar between non-treated and UA-treated animals (*p* = 0.5845 and *p* = 0.5243, respectively). Likewise, there were no significant treatment differences in ROS production from circulating (3E) and lung infiltrating (3F) PMNs (*p* = 0.6251 and *p* = 0.5993, respectively).

Irrespective of cell type or location, we did not observe any treatment differences in ROS production, which suggests altered IL-17 signaling is not impacting the oxidative burst functionality of innate immune cells following MH infection. We also noted that UA treatment did not alter the frequencies of host cells infiltrating the BAL fluid; however, UA-treated animals tended to have a lower MH burden (*p* = 0.0605) compared to non-treated animals. Therefore, we speculate that altered IL-17 signaling may be altering other aspects of the immune response to limit bacterial colonization and pathogenesis following infection.

### Prophylactic UA Treatment Modulates Inflammatory Signaling Following MH Infection

The immune response to MH infection includes production of the chemokine IL-8 (neutrophil chemotactic factor, or CXCL-8) and the inflammatory cytokine IL-6. IL-6 binds to surface receptors of other immune cells to induce STAT3 signaling and subsequent activation of the RORC gene; RORC encodes the transcription factor (RORγt) for IL-17A production. IL-17A subsequently acts in a positive feedback loop to induce further upregulation of IL-8. Therefore, we next chose to investigate the impact of UA treatment, and its suppression of IL-17A signaling, on this inflammatory pathway during MH infection.

**Figure 4:**
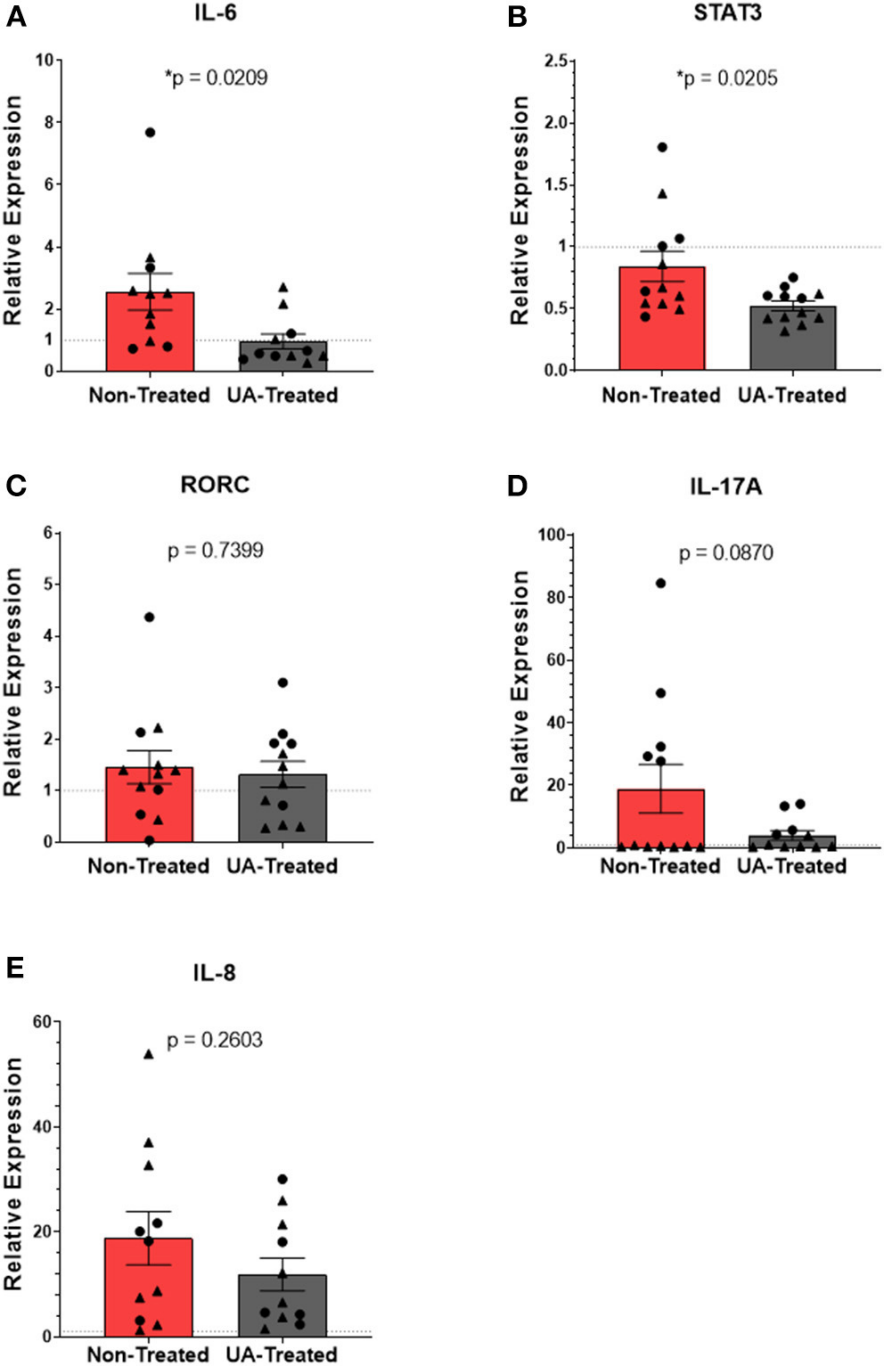
Prophylactic UA treatment modulates inflammatory IL-17A signaling in the lungs following MH infection. Samples of lung tissue were collected at necropsy and stored in RNAlater solution for qPCR analysis of **(A)** IL-6, **(B)** STAT3, **(C)** RORC, **(D)** IL-17A, and **(E)** IL-8 mRNA expression; individual animal data from F2019 has been represented as a circle (•), while S2020 animal data has been represented as a triangle (▴). The relative mRNA expression in pneumonic lung tissue from non-treated and UA-treated animals was compared to healthy lung tissue from control animals (F2019 group) for both studies. The average relative expression (RE) ± the SEM for each treatment group across both studies is depicted in **(A–E)**. Outliers were identified using the ROUT method (Prism v9.1.0), and the data were analyzed using a standardized unpaired *t*-test (*p < 0.05). **(A)** Non-treated *n* = 11, UA-treated *n* = 11; **(B)** non-treated *n* = 12, UA-treated *n* = 12; **(C)** non-treated *n* = 12, UA-treated *n* = 12; **(D)** non-treated *n* = 12, UA-treated *n* = 11; **(E)** non-treated *n* = 11, UA-treated *n* = 11.

Figures 4A–E show the relative mRNA expressions of genes associated with the IL-17A signaling pathway; each gene's RE was determined using the 2^−ΔΔCT^ method by comparing ΔCTs from pneumonic lung tissue to healthy control lung tissues obtained during the F2019 study. Figure 4A shows significantly (*p* = 0.0209) higher IL-6 expression in the lungs of non-treated animals compared to UA-treated animals following MH infection. As seen in Figure 4B, UA treatment also significantly (*p* = 0.0205) down-regulated STAT3 expression compared to non-treated animals. Intracellular STAT3 signaling has been shown to transcriptionally regulate other immune activating and inflammatory genes (29, 30). Interestingly, the relative mRNA expression for the RORC gene (Figure 4C) does not appear to differ (*p* = 0.7399) between treatment groups; since this gene is tightly regulated at multiple levels its expression may be more immutable. Downstream from the RORC gene, IL-17A expression (Figure 4D) was reduced (*p* = 0.0870) in UA-treated animals compared to non-treated animals. However, IL-17A may not be the only neutrophil activating signal being differentially expressed during MH infection. Subsequently, we investigated the impact of the IL-17A inhibitor (UA) on IL-8 expression (Figure 4E, *p* = 0.2603), but did not observe any significant differences in treated compared to non-treated animals.

### Prophylactic UA Treatment Alters Expression of Innate Defense Genes in Response to MH Infection

**Figure 5:**
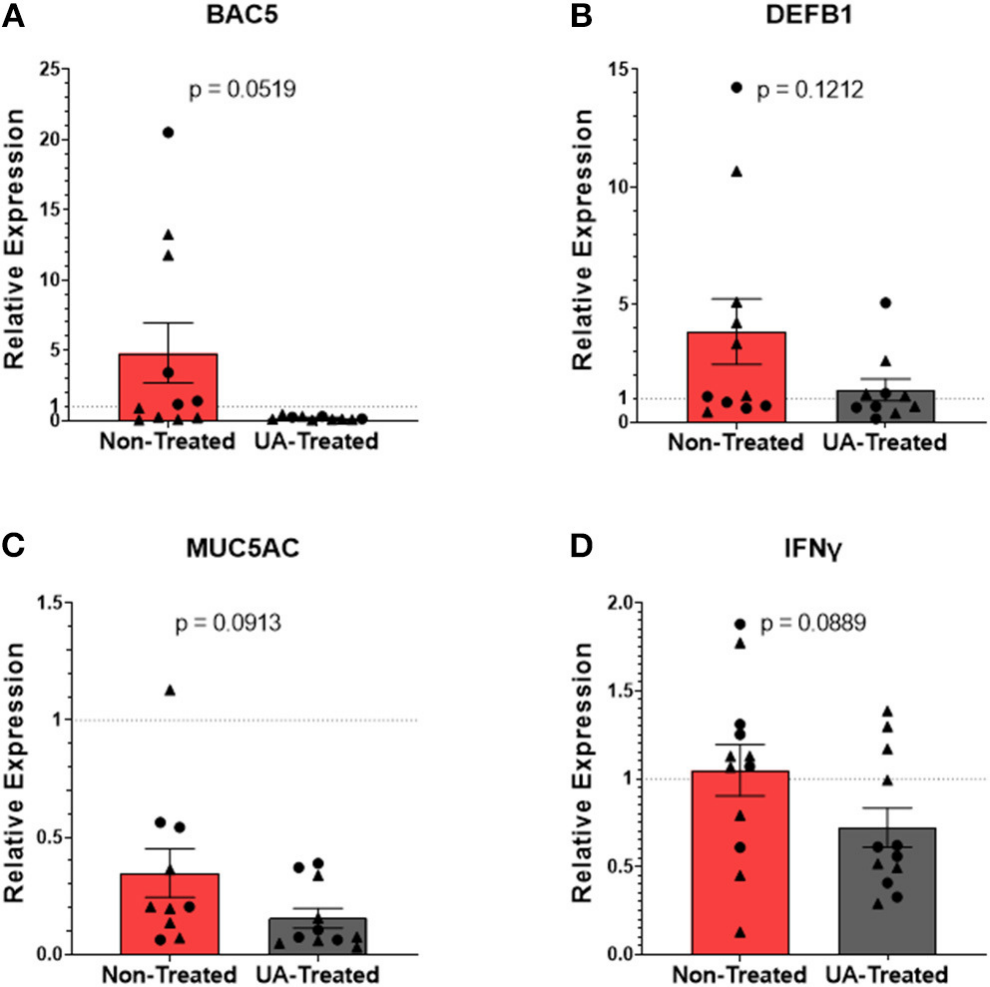
Prophylactic UA treatment alters expression of innate defense genes in the lungs following MH infection. Samples of lung tissue were collected at necropsy and stored in RNAlater solution for qPCR analysis of **(A)** BAC5, **(B)** DEFB1, **(C)** MUC5AC, and **(D)** IFNγ mRNA expression; individual animal data from F2019 has been represented as a circle (•), while S2020 animal data has been represented as a triangle (▴). The relative mRNA expression in pneumonic lung tissue from non-treated and UA-treated animals was compared to healthy lung tissue from control animals (F2019 group) for both studies. The average relative expression (RE) ± the SEM for each treatment group across both studies is depicted. Outliers in the data sets were first identified using the ROUT method (Prism v9.1.0), and the data were analyzed using a standardized unpaired *t*-test (^*^p < 0.05). **(A)** Non-treated *n* = 11, UA-treated *n* = 10; **(B)** non-treated *n* = 11, UA-treated *n* = 10; **(C)** non-treated *n* = 10, UA-treated *n* = 11; **(D)** non-treated *n* = 12, UA-treated *n* = 12.

Neutrophilic granules contain a multitude of antimicrobial peptides (AMPS) that can be weaponized against invading pathogens. In addition to its role in the inflammatory cascade, IL-17A also plays an important role in activating neutrophil expression of AMPS and other innate defense molecules (31, 32). Figures 5A–D show the relative mRNA expressions of genes associated with the host's innate defenses; each gene's RE was determined using the 2^−ΔΔCT^ method by comparing ΔCTs from pneumonic lung tissue to healthy control lung tissues obtained during the F2019 study. Bactenecin-5 (BAC5) is an AMP found in bovine neutrophils that is effective at inhibiting the growth of and killing Gram-negative bacteria (33), and its expression is controlled under the BAC5 (CATHL2) gene. Interestingly, relative expression of BAC5 (Figure 5A) tended to be downregulated (*p* = 0.0519) in the lungs of UA-treated animals compared to those of untreated controls. β-defensin 1 is an AMP capable of permeabilizing membranes and inducing neutrophil extracellular traps (NETs) to control bacterial pathogens (34). Its expression is regulated under the DEFB1 gene in epithelial cells that line the respiratory tract. In Figure 5B, we do not see a significant difference in the expression of lung DEFB1 (*p* = 0.1212) between non-treated and UA-treated animals. MUC5AC is another innate defense gene commonly expressed in the lungs during respiratory infections, and its expression leads to the production of gel-forming mucins that can trap extracellular pathogens. However, the buildup of these mucins can block airways and contribute to lung congestion, so managing mucin production is important for both mucociliary function and pathogen clearance (3, 35). Figure 5C shows that the relative expression of MUC5AC is down regulated in non-treated and UA-treated animals alike, but UA-treated animals tend to express lower levels compared to untreated controls (*p* = 0.0913), which may be indicative of improved infection-resolution in their lungs. Interferon-γ (IFNγ) signaling can play an overlapping role in both the induction of AMPs and inhibition of some mucins. As seen in Figure 5D, the relative expression of IFNγ tends to be reduced in UA-treated calves compared to control calves (*p* = 0.0889), and is thus not likely a driving factor for the observed increase in AMP production.

### Prophylactic UA Treatment May Influence Lung Tissue Remodeling Following MH Infections

Matrix metalloproteinases (MMPs) are produced to degrade the extracellular matrix and allow for more efficient leukocyte infiltration while clearing away damaged and necrotic tissues. MMP9 (gelatinase B, a type IV collagenase) has been implicated in lung tissue damage following MH infections; its release by bovine neutrophils during degranulation can exacerbate inflammatory signaling through the induction of both IL-1 and IL-8. Macrophage production of the tissue inhibitor of metalloproteinase 1 (TIMP-1) can be upregulated to offset some of the inflammatory and degradative effects of MMP9, and it is thought that an imbalance of these two proteins may play a role in disease progression (29, 36). Since inflammatory signaling has a profound effect on tissue remodeling processes, we speculated that inhibited IL-17 signaling may alter the balance of MMP production and inhibition.

**Figure 6:**
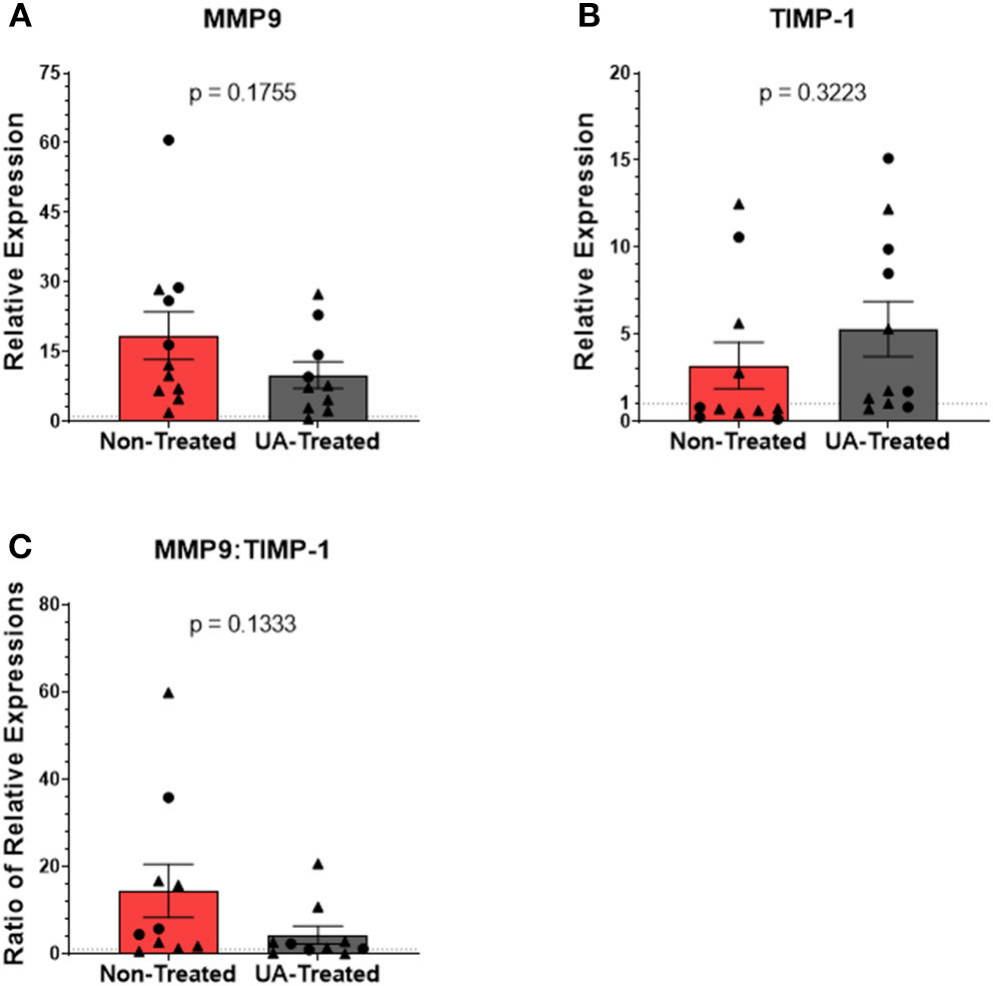
Prophylactic UA treatment alters matrix metalloproteinase activity in the lungs following MH infection. Samples of lung tissue were collected at necropsy and stored in RNAlater solution for qPCR analysis of **(A)** MMP9 and **(B)** TIMP-1 mRNA expression; individual animal data from F2019 has been represented as a circle (•), while S2020 animal data has been represented as a triangle (▴). The relative mRNA expression in pneumonic lung tissue from non-treated and UA-treated animals was compared to healthy lung tissue from control animals (F2019 group) for both studies. Graphs depict the average relative expression (RE) ± the SEM for each treatment group across both studies. Outliers in the data sets were first identified using the ROUT method (Prism v9.1.0), and the data were analyzed by standardized unpaired *t*-test (^*^p < 0.05). **(A)** Non-treated *n* = 11, UA-treated *n* = 10; **(B)** non-treated *n* = 11, UA-treated *n* = 11). **(C)** Depicts the ratio of MMP9 to TIMP-1 RE for each individual animal. Data represent mean RE ratios ± the SEM for each treatment group.

Relative expression levels of MMP9 (Figure 6A) are not significantly different (*p* = 0.1755) between non-treated and UA-treated animals, although the mean expression of MMP9 is higher in control animals (non-treated = 18.44; UA-treated = 9.95). The relative expression of TIMP-1 (Figure 6B) did not differ (*p* = 0.3223) between UA-treated animals and non-treated animals. However, only 4/11 control animals have a relative expression value >1, indicating that most of these animals have downregulated TIMP-1 expression relative to healthy lung samples. Meanwhile, 8/11 UA-treated animals have a RE value >1, indicating an upregulation of TIMP-1 compared to healthy lungs. TIMP-1 directly antagonizes the degradative capabilities of MMP9 in a 1:1 ratio (36); therefore, comparing MMP9 expression to TIMP-1 expression provides insight into the state of tissue repair occurring in the lungs following MH infections. Figure 6C shows the ratio of MMP9 to TIMP-1 relative expressions for each animal, and this comparison highlights an interesting tendency (*p* = 0.1333) between UA-treated and non-treated animals, with UA-treated animals having a lower mean ratio and clustering more tightly than non-treated animals. Considering the antagonistic functionality of MMP9 and TIMP-1, these data suggest that our control animals may still be actively remodeling lung tissue to combat infection, while the UA-treated animals with altered IL-17 signaling may be resolving the lung environment to repair functionality.

## Discussion

Inflammatory cytokines, like IL-17A, are thought to contribute to lung congestion by orchestrating the mobilization of neutrophils, inducing the production of ROS and MMPs, and activating the innate defenses from cells lining the respiratory tract. Production of IL-17A, and the subsequent IL-17A-driven inflammation, is regulated by the transcription factor RORγt (12, 13). The small molecule inhibitor, UA, is reported to be a specific and highly effective agonist of RORγt (12). We believe this is the first time the drug has been implemented in a bovine model, and our *in vitro* (Figure 1B) and *in vivo* (Figure 4D) results underpin UA's inhibitory effects on IL-17A production. Although applications of UA treatment are being investigated clinically, the drug still has limitations in its therapeutic potential. Initial reports have implied that UA treatment is specific for inhibiting IL-17A production, but more recent reports show that treatment effects may not be precise. A recent publication from Zhang et al., has shown that UA is also a potent agonist of peroxisome proliferator-activated receptor γ (PPARγ) in experimental models of multiple sclerosis (37). Other reports have determined that UA treatment suppresses the phosphorylation of STAT3 and JAK2, which further explains the drug's capacity to inhibit Th17 differentiation (13, 38). Despite not having looked at phosphorylation levels, we did see a significant reduction in STAT3 expression in the lungs of UA-treated calves (Figure 4B) consistent with these reports. Thus, although UA was a useful tool to impair IL-17 signaling in our studies, its effects may not be specific only to IL-17A production.

There is a significant degree of variability amongst animal trials utilizing experimental MH infection. One study used 4.10 × 10^7^ CFUs of MH inoculum to induce clinical signs similar to a natural infection (39). Meanwhile, other reports have employed considerably higher doses (4.4 × 10^11^ CFUs) (36). Considering the variability in disease outcomes and infection doses, we selected an inoculum of 2–4 × 10^8^ total MH CFUs. While this inoculation strategy did produce clinical MH infection, the resulting disease was severe in some animals (mostly in the F2019 study), which may have limited our ability to discern differences between treatment groups. Although some of these differences may be attributed to natural variations between animals, the F2019 animals also developed a concurrent infection with *Pasteurella multocida* (PM). Subsequently, F2019 animals had higher MH burdens than S2020 animals (Figure 3A), and lung cultures from F2019 animals also revealed the presence of PM in both control and UA-treated animals. Importantly, the F2019 animals were evaluated by thoracic ultrasonography the day prior to infection (Porter et al., manuscript under review) and their lungs were clear of consolidation or pleural defects that would indicate a prior or ongoing bacterial infection. This suggests that F2019 animals were either in the early stages of PM infection, or possibly that our intratracheal MH infection created a window of susceptibility for PM infection. We speculate that the increased pathogenesis from PM co-infection may have limited our ability to discern more significant treatment effects, and future studies should consider the balance between inducing pathogenesis and over-challenging beyond the immunomodulatory capabilities of UA.

MH infection is characterized by lung consolidation with infiltrating neutrophils found in the lung exudate (40). There is also a well-established role for IL-17A inflammation in driving the production of neutrophil chemoattractants and activating innate defenses (32, 41). We have previously reported that MH infection induces IL-17A cytokine production in the lungs (8). Ergo, we hypothesized that altering IL-17A-driven inflammation during MH infection would reduce the presence or activation of infiltrating leukocytes in the lung. Interestingly, we did not observe any changes in the frequency of neutrophils isolated from the BAL fluid of calves treated with the IL-17A inhibitor (Figure 3B). Although the UA-treated animals tended to have lower IL-17A expression in their lung tissues (Figure 4D), we observed no differences in the expression of IL-8 (Figure 4E) (42). It is plausible that neutrophil infiltration was not impacted by altered IL-17 signaling due to IL-17A-independent production of IL-8. To this point, LPS (shed from MH) and damage-associated molecular patterns (induced from ROS and leukotoxins) can directly stimulate alveolar macrophages and epithelial cells to produce IL-8 in the absence of IL-17 signaling (42–44). Others have demonstrated that LPS and flagellin can activate TLR4 and TLR5 signaling cascades in bronchial epithelial cells (through MAPK and NF-κB pathways) to release of large quantities of TNFα, IL-6, and IL-8 (43), which can synergize to recruit and activate neutrophils and macrophages apart from IL-17 signaling (31, 42, 43, 45).

Inflammatory signaling following MH infection is known to induce tissue remodeling by matrix metalloproteinases. MMP9 is an important gelatinase which contributes to lung tissue damage during MH infection (36). Initially, MMP9 production causes degradation of the extracellular matrix, allowing for increased immune cell infiltration. However, prolonged production of MMP9 leads to degradation of the basement membrane of the lung epithelia, which compromises the architecture and barrier functions within the airways, resulting in consolidated lung lesions (36, 46). TIMP-1 is the conjugate inhibitor to MMP9, and its expression is induced to temper the tissue degradation spurred on by inflammatory signaling (36). The inverse ratio between these two proteins is helpful in assessing the state of lung remodeling following acute damage or infections (47, 48). In our study, UA-treated animals had a lower (more inhibitory) expression ratio of MMP9:TIMP-1 (mean expression ratio of 4.287) compared to the non-treated control calves (mean expression ratio of 14.437). Although the treatment difference between the ratios of MMP9:TIMP-1 was not statistically significant, it suggests that lung remodeling and tissue repair may be expedited when inflammatory IL-17 signaling is altered.

## Conclusion

Herein, we have shown that inflammatory IL-17 signaling following MH infection plays a dual role in pathogen clearance and immunopathogenesis which may adversely affect disease resolution. We have observed that animals receiving an IL-17A inhibitor tended to have lower lung pathology scores and reduced bacterial colonization in the lungs compared to control animals. Furthermore, blocking the IL-17A signaling pathway resulted in altered expression of inflammatory and innate defense genes in the lungs, and impacted the expression of genes related to tissue remodeling in the lungs following MH infection. Taken together, our results suggest that inflammatory IL-17 signaling plays an important role in MH infection and BRDC pathogenesis, and further investigations into this pathway may offer new therapeutic intervention strategies for respiratory infections.

## Data Availability Statement

The original contributions presented in the study are included in the article/Supplementary Material, further inquiries can be directed to the corresponding author/s.

## Ethics Statement

The animal study was reviewed and approved by Iowa State University Institutional Animal Care and Use Committee.

## Author Contributions

JS, BC, RB, and JM designed the study. JS and BC collected and analyzed the data. JS and JM wrote the manuscript. All authors reviewed the manuscript.

## Funding

Early *in vitro* work was supported through funds from the NIH Grant R21 AI127895. The animals and work detailed in this manuscript were funded by the USDA-NIFA Grant 2018-06904 to JM. The funders had no role in study design, data collection and analysis, decision to publish, or preparation of the manuscript.

## Conflict of Interest

The authors declare that the research was conducted in the absence of any commercial or financial relationships that could be construed as a potential conflict of interest.

## Publisher's Note

All claims expressed in this article are solely those of the authors and do not necessarily represent those of their affiliated organizations, or those of the publisher, the editors and the reviewers. Any product that may be evaluated in this article, or claim that may be made by its manufacturer, is not guaranteed or endorsed by the publisher.
