# Supplementary material for: The Effects of Ursolic Acid Treatment on Immunopathogenesis Following Mannheimia haemolytica Infections
Source: Front Vet Sci. 2021 Nov 18;8:782872. doi: 10.3389/fvets.2021.782872 (PMC8637451; doi:10.3389/fvets.2021.782872)
Supplement: Supplementary file 1 [file Image_1.pdf]

Supplementary Material

1 Supplementary Figures and Tables

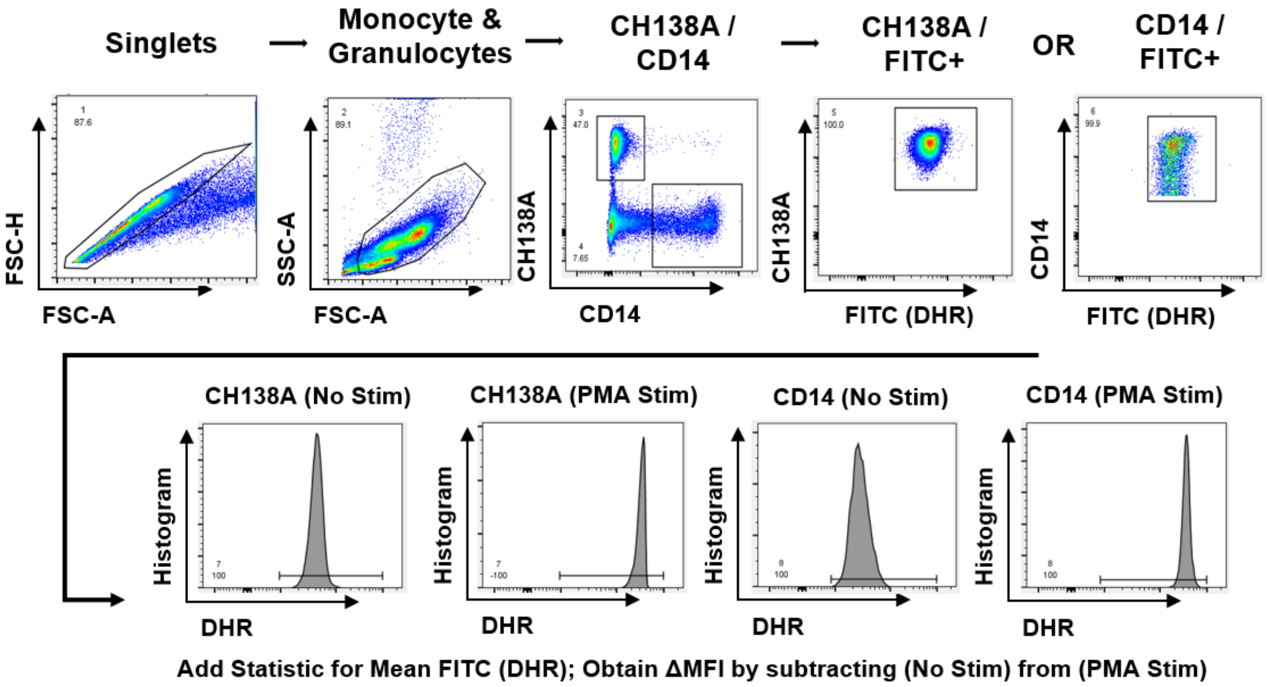

Supplementary Figure 1. Gating strategy for FACS analysis of Dihydrorhodamine 123 (DHR) Reactive Oxygen Species (ROS) Assay. Whole blood and BAL fluid were collected at 4 dpi and cultured with or without PMA stimulation to determine the oxidative burst potential of monocytes and granulocytes using a flow cytometry based DHR Assay. Cells were surface stained with monoclonal antibodies to detect monocytes (anti-bovine CD14) and granulocytes (anti-bovine CH138A) and loaded with DHR. Oxidative burst activity was analyzed based on the conversion of fluorescent rhodamine 123 (analyzed in the FITC channel). The mean fluorescence intensity (MFI) of DHR was determined by gating on singlets, monocytes (CD14+) and granulocytes (CH138A+), and analyzing FITC expression. The ΔMFI of DHR for each cell population in individual animals was calculated by subtracting the DHR MFI of non-stimulated cells from the DHR MFI of PMA stimulated cells. These DHR ΔMFI values were compiled, charted, and analyzed for statistical significance in Prism v9.1.0 (GraphPad Software, Inc.).
